# Phytogenics in Ginger, *Origanum vulgare*, and *Syzygium aromaticum* and Their Potential as a Feed Additive against *Clostridium perfringens* in Broiler Production

**DOI:** 10.3390/ani13233643

**Published:** 2023-11-24

**Authors:** Gilmour Valdez, Lie-Fen Shyur, Sheng-Yang Wang, Shuen-Ei Chen

**Affiliations:** 1 Department of Animal Science, National Chung Hsing University, Taichung 40227, Taiwan; gilmour.gleah@gmail.com; 2 San Mateo Campus, Isabela State University, San Mateo 3309, Isabela, Philippines; 3 Agricultural Biotechnology Research Center, Academia Sinica, Taipei 15201, Taiwan; jaclyn@gate.sinica.edu.tw; 4 Department of Forestry, National Chung Hsing University, Taichung 40227, Taiwan; 5 i-Center for Advanced Science and Technology (iCAST), National Chung Hsing University, Taichung 40227, Taiwan; 6 The iEGG and Animal Biotechnology Center, National Chung Hsing University, Taichung 40227, Taiwan; 7 Innovation and Development Center of Sustainable Agriculture (IDCSA), National Chung Hsing University, Taichung 40227, Taiwan

**Keywords:** necrotic enteritis, phytogenics, *Clostridium perfringens*, broilers, inflammation

## Abstract

**Simple Summary:**

Phytogenics such as ginger, wild marjoram, and cloves, along with their active components, have been shown to be effective and safe in animal production and thus serve as potential substitutes that could replace antibiotic growth promoters. This review delineates their common mechanisms and summarizes the results of recent studies on the growth performance of poultry production—with emphasis on broilers—to combat necrotic enteritis.

**Abstract:**

Necrotic enteritis is a devastating disease in chickens mainly caused by *Clostridium perfringens*—particularly, Net-B toxin-producing strains. In order to combat necrotic enteritis in broiler production, natural growth promoters, as well as anti-inflammatory and non-antibiotic remedies, were developed for anti-microbial resistance due to its status as a global pandemic. Herein, phytogenic ginger, wild marjoram, and cloves were reviewed as potential alternatives to antibiotics for their anti-microbial functions. These phytogenics contain active ingredients that efficiently modulate the immune response and improve intestinal morphology and overall growth performance, even under stress and infection conditions. Most of the beneficial effects can be attributed to their anti-inflammatory functions, primarily the inhibition of the NF-κB and MAPK pathways. Phytogenics and their active ingredients represent potential substitutes for antibiotic growth promoters, further serving as anti-microbial remedies in the treatment of birds with infections.

## 1. Introduction

Effective and practical alternatives to replace antibiotic growth promoters (AGPs) have become essential and urgent, since the problem of anti-microbial resistance (AMR) has greatly impacted health in both humans and animals. The issue of AMR is considered a global pandemic by the World Health Organization [1]. The use of AGPs also has the advantage of preventing infectious diseases, such as avian *Colibacillus* and *Salmonellosis*, which could result in a wide range of illnesses and poor growth efficiency, leading to a significant increase in production costs [2]. However, due to the concern that using AGPs increases resistance in bacteria, alternative strategies have been extensively explored to reduce dependence on AGPs in poultry production [3]. However, a decline in the usage of AGPs in broiler production increases the occurrence of cost-causing diseases, such as necrotic enteritis (NE), a devastating intestinal illness in chickens, which has detrimental effects on the poultry industry and can lead to great profit loss due to its high mortality rate [4].

Necrotic enteritis usually occurs in broilers around 4 weeks after hatching [5]. A variety of factors contribute to NE, of which *Clostridium perfringens* (CP), a Gram-positive spore-forming anaerobe, is the major causative factor [5]. Since CP is a part of the intestinal microbiota of birds, its presence might not be a suitable indicator of NE. In healthy birds, more than 10^5^ CP/g of their intestinal contents are maintained in the intestine. However, it is estimated that up to 10^9^ CP/g may cause overt NE illness in broilers [6].

Clinical signs of NE are indistinctive, and its duration is very short; normally, infected chicks only show severe depression, and they suffer acute death within hours. In a very severe outbreak, there is a sudden increase in flock mortality. In subclinical necrotic enteritis (SNE), chronic damage to the intestinal mucosal layer tends to impair digestion and absorption, thus leading to poor growth performance [7]. Gross lesions are usually observed in the small intestine, which demonstrates a thinner wall and is filled with gas. In overt clinical NE cases, mucosal necrotic lesions are characterized as spreading in a large part along the small intestine, with a yellow-brown pseudomembrane [8]. Subclinical cases typically demonstrate ulcers with faint-colored exudate materials adhering to the depressed mucosal surface [9]. Moreover, SNE impacts production efficiency and profitability. In contrast to healthy birds, birds with SNE are estimated to undergo a 12% reduction in body weight and a 10.9% increase in FCR [10]. According to calculations, NE costs the industry USD 6 billion yearly [11].

Some predisposing factors, including altered gut microbiota, substantial epithelial surface injury, and immunosuppression, favor CP colonization and proliferation in the intestine [12]. Genetic selection for rapid growth in broilers makes current broiler strains more susceptible to NE [13]. Additional risk factors, including vaccination, such as that for infectious bursal disease (IBD); feed formula; and environmental stress, including ventilation, litter moisture, thermal stress, and flock density, also contribute to the prevalence of NE outbreaks [5,14]. Numerous studies suggest that CP alone exerts minor damage to the intestine unless it is connected to detrimental predisposing factors, coccidia infection, and a high protein ration in diets, mostly formulated using fishmeal. A high portion of fishmeal proteins in the diet as well as coccidiosis, particularly via *Eimeria tenella* infection, predispose the environment in birds’ guts to NE development by altering the gut microbiota, thus favoring CP proliferation [15]. When birds are infected with CP, the short-chain fatty acids (SCFAs) produced by coccidia and a high-protein fishmeal significantly alter the ecology in the intestine, leading to a favorable environment for NE progression [16]. The three variables, high-protein diet, coccidia infection, and CP, interact synergistically, and NE cannot be caused by a lack of one of them. For instance, high-protein fishmeal, without the presence of coccidia and CP infection, still improved body weight gain, and no signs of NE were observed [16], while in the presence of co-infection with CP and *Emeria*, the portion of beneficial bacteria in the ileal microbiota declined significantly, thus greatly favoring the development of NE [17]. A significant contribution by a non-toasted bean diet also promoted the occurrence of SNE in broilers, which exhibited remarkable lesions in the intestine, as the non-toasted soybean diet is enriched with high trypsin inhibitor content, which prolongs the retention of digesta in the gut and thus favors CP proliferation [18]. Feed formulations, such as a wheat-based diet, contain a high portion of non-starch polysaccharides (NSPs), which increase digesta viscosity, prolong its retention, and favor CP proliferation and NE development. These elements contribute to the affected broilers’ gut health and, eventually, growth performance [19]. Understanding the underlying predisposing factors can help in the development of preventive strategies to minimize the occurrence of NE in broiler flocks.

**Figure 1:**
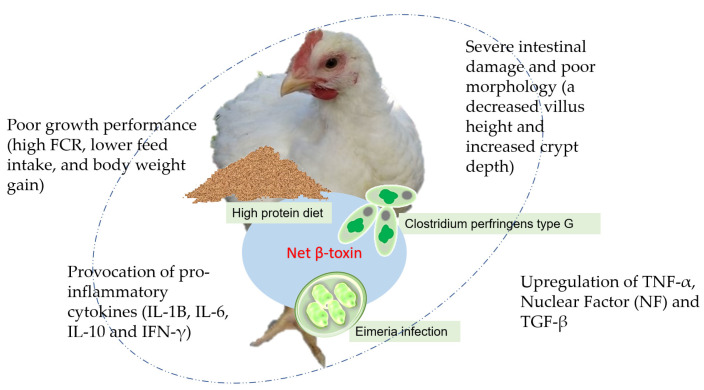
The possible mechanisms of a high-protein diet, coccidia, and *Clostridium perfringens* infection causing necrotic enteritis in chickens.

Strains of *C. perfringens* that produce the Net B-toxin are more likely to cause overt NE. Net B-producing CP, of type A strains, are highly associated with the development and severity of NE in affected individuals [20,21]. Figure 1 summarizes how predisposing factors cause NE in broiler chickens. Recent studies reported that the Net B-toxin can be detected in the type G strain and has a modest propensity to induce NE, regardless of the presence of predisposing factors such as coccidiosis, high-protein diet, and others that can change gut environments [22]. The type G strain of the Net B-toxin even determines the virulence of CP and promotes the severity of NE when CP and coccidia infections coexist [23].

The growth performance of broilers is remarkably impacted by NE. It lowers body weight gain and feed intake, increases feed conversion rate (FCR), and raises mortality rates [24,25]. Poor growth performances are associated with changes in gut microbiota, injury, immune response, and morphology [5,14].

Birds with NE were noted as suffering from intestinal inflammation, as shown by the upregulation of interleukin-6 (IL-6), IL-10, IL-1β, and the transforming growth factor (TGF-β), in addition to an increased TNF-α receptor exacerbating the damage on the gut epithelial linings [19,25]. Immune-regulatory IL-10 is essential to control the development of the immune response, whose expression is greatly upregulated in the intestines of birds with NE [26]. Additionally, the intricate relationship between coccidia and CP can enhance the upregulation of IL-8 to activate heterophils and their recruitment to the site of infection, both of which are essential for preventing bacterial invasion but may prolong the inflammatory state, leading to tissue swelling and necrosis. The anti-inflammatory IL-10, on the other hand, is produced to prevent an over-inflammatory response from CP infection. LITAF (lipopolysaccharide-induced TNF factor) and IL-15 are crucial for boosting immune responses against pathogens [27]. *C. perfringens* challenge induces the activation of Toll-like receptor (TLR)-4 and TLR-2, and downstream nuclear factor-kappa B (NF-κB) and JAK3 (Janus Kinase 3) pathways, leading to the provocation of intestinal pro-inflammatory cytokines [25], in which mitogen-activated protein kinase (MAPK) pathways primarily respond to CP infection and mediate the production of pro-inflammatory factors following inflammation [28].

These detrimental impacts of NE on broiler production manifest the urgent requirement for efficient preventative interventions, particularly to meet the need for a sustainable and natural system for poultry production, which is a global trend nowadays. Phytogenics have been shown to have anti-microbial, antioxidant, anti-inflammatory, and even growth-promoting effects on domestic animal production, making them one of the most promising options for the replacement of AGPs [29]. These plant-based compounds possess anti-microbial abilities without a risk of antibiotic resistance [30]. Their multifaceted properties render them an attractive solution to not only combat NE but also other pathogens [31]. Additionally, their antioxidant properties potentially contribute to gut health by reducing oxidative stress and even inflammation [32]. By incorporating phytogenics into a feed formula, poultry farmers can enhance growth performance and ensure a sustainable production system [33].

Embracing these innovative, phytogenic-based approaches not only addresses the urgent need for preventative interventions but also aligns with the global movement towards reducing antibiotic usage in animal production. In this overview, typical plant extracts from ginger, *Origanum vulgare*, and cloves, as well as their bioactive components, are highlighted—particularly for their antibacterial, growth-promoting, and immunomodulatory functions that have been validated in feeding trials for practical use. Bioactive compounds are also discussed in order to provide a complete understanding of their underlying mechanisms. These phytogenics and their active ingredients may share common mechanisms and functions as growth promoters used in a poultry diet. This review provides information for future studies and developments using these phytogenics and their active ingredients as preventive interventions or remedies to treat birds infected with various bacteria—especially against NE by the *C. perfringens* infection reviewed herein. The antibacterial effects of the plant extracts and their bioactive components against other specific bacteria are also noted in each table.

## 2. Effects of Plant Extracts and Their Bioactive Compounds

### 2.1. Ginger and Its Active Compounds

Ginger (*Zingiber officinale*) has a long history of being used in traditional medicine due to its therapeutic properties, which have been recognized for centuries [34]. Its plant parts, extracts, and active compounds are known for their anti-inflammatory, antioxidant, and anti-microbial functions, making it a valuable natural remedy not only for humans but also for animals (Table 1) [35,36,37,38,39,40,41,42,43,44,45].

Ginger rhizomes contain various phytochemicals that are rich in antioxidant and antibacterial functions [43]. Hydro-alcoholic extracts from dried ginger rhizomes are effective against *Staphylococcus aureus*, *Pseudomonas aeruginosa*, and *Listeria monocytogenesis* [36]. Furthermore, its ethanolic extracts contain anti-microbial compounds against *E. coli*, *Salmonella* typhi, and *Bacillus subtilis* [37]. Fresh ginger is enriched with gingerol (6-gingerol, 8-gingerol, and 10-gingerol), which has been shown to possess anti-gastrointestinal cancer effects [38]. Moreover, ginger roots (dry or fresh) and their active compounds (6-gingerol as well as 4-, 5-, 8-, 10-, and 12-gingerols) have anti-fungal, antibacterial, anti-inflammatory, analgesic, and immunomodulatory effects [39]. 6-gingerol extracted from ginger rhizomes also possesses anti-inflammatory [40] and anti-colitis effects [41]. Gingerol extracted from rhizomes are effective against *E. coli*, *Salmonella typhi*, and *Bacillus subtilis* [42].

When ginger is dried, most of its bioactive gingerols are converted to shogaols. This compound is effective against both Gram-positive and -negative bacteria [43] and has anti-inflammatory and antioxidant functions [44]. *Staphylococcus aureus* was found to be sensitive to gingerenone-A and shogaol essential oil from ginger extracts [45], while a mixture of extracts from ginger was reported to exert anti-clostridial effects in vitro [46].

**Table 1:** Ginger extract plant parts, active compounds, and biological properties.

Bioactive Components or Extracts	Parts of the Plant for Extraction	Functional Property	References
Whole phytochemical extracts	Dried rhizomes	Antioxidant and antibacterial activities	[35]
Hydro-alcoholic extracts	Dried rhizomes	Antibacterial effects against *Staphylococcus aureus*, *pseudomonas aeruginosa*, and *Listeria monocytogenes*	[36]
Ethanolic extracts	Fresh ginger rhizomes	Anti-microbial effects against *E coli*, *Salmonella* typhi and *Bacillus subtilis*	[37]
Gingerol (6-gingerol, 8-gingerol, and 10-gingerol)	Rhizomes	Anti-cancer activities	[38]
Ginger oil (6-gingerol as well as 4-, 5-, 8-, 10-, and 12-gingerols)	Ginger roots	Anti-fungal, antibacterial, anti-inflammatory, analgesic, and immunomodulatory effects	[39]
6-gingerol	Rhizomes	Anti-inflammatory activities	[40]
6-gingerol	Rhizomes	Anti-colitis activities	[41]
Gingerol	Rhizomes	Effective against *E. coli*, *Salmonella* typhi and *Bacillus subtilis*, with anti-fungal effects	[42]
6-shogaol	Dried ginger rhizomes (gingerols converted to shogaols)	Anti-microbial effects against Gram-positive and -negative bacteria	[43]
6-Shogaols	Dried rhizomes	Anti-inflammatory, antioxidant properties	[44]
Gingerenone-A and shogaol	Rhizomes	Anti-microbial effects against *Staphylococcus aureus*	[45]

#### 2.1.1. Effects of Ginger on the Growth Performance of Broiler Chickens

A dietary supplementation of ginger extracts or ground ginger has been shown to improve growth performance in broilers [47]. The promotion of feed intake was even higher than those with a control diet containing antibiotics as a growth promoter [48]. Ground fresh ginger is apparently more effective than dried ginger in improving growth performance [49]. Some studies demonstrated that the improved growth performance by supplemental ginger is only limited to body weight gain, while other parameters, such as FCR, remain unchanged [50]. Moreover, broilers receiving supplemental ginger extracts were more resistant to heat stress and showed improved growth performance, including FCR [51]. Gingerol is also effective in improving body weight gain and FCR in birds suffering from heat stress [52].

The inclusion rate of ginger extracts varies in its effects on growth performance. A high inclusion at a 0.6% level did not improve growth performance in broilers, even decreasing the final body weight, but at levels of 0.2 and 0.4%, it significantly improved feed intake and FCR [53]. This is in contrast to another report stating that the inclusion of ginger root powder at a 0.6% level increased body weight gain and improved FCR and production indices [54]. Higher inclusion rates of ginger apparently have no negative effects on boiler performance, as levels of the inclusion of ginger extracts as high as 6% exerted no negative effects on body weight gain and even improved FCR [55]. The inclusion of ginger root powder was optimized at 7.5 g/kg feed to improve body gain weight [56], while a 1.5% inclusion had no effects on growth performance [57]. Ginger supplementation may exert beneficial effects for the prevention of necrotic enteritis. As mentioned above, coccidiosis must co-exist with a CP infection in order to induce overt NE development [15]. Supplemental ginger has been confirmed to mitigate the detrimental effects of *Eimeria* infection in broilers and improve the overall growth performance [58]. Therefore, ginger supplementation may be practically used to prevent NE occurrence in broiler production. This improved growth performance was partially attributed to a better intestinal histology, including higher villus lengths and lower crypt depths for a more efficient absorption process [47,48,53].

#### 2.1.2. The Anti-Inflammatory and Immunomodulatory Properties of Ginger and Its Active Compounds

**Figure 2:**
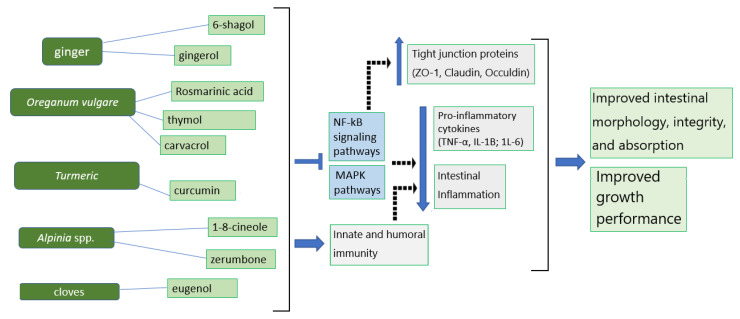
The mechanisms of phytogenics and their active ingredients for the suppression of inflammation and improvement of intestinal integrity and growth performance in broiler chickens. 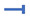
—inhibitory effects; 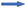
—stimulatory effects; 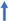
—upregulation; 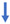
—downregulation.

Despite no studies on ginger extracts and their active compounds specifically conducted in broilers with respect to NE development, emerging evidence has suggested that ginger and its active ingredients may produce anti-inflammatory responses against NE development (Figure 2). 6-shogaol (6-SG), as one of the active components of ginger, was found to exert an immuno-protective effect by inhibiting phosphatidylinositol-3-kinase/Akt and NF-κB signaling [39], leading to an alleviated downregulation of intestinal claudin-1 and -2 by TNF-α [59]. Other studies in a rat model also confirmed that 6-gingerol prevents intestinal inflammation, including TNF-α, IL-1β, and IL-6 provocation, by inhibiting p38 MAPK and thereby protecting the intestinal barriers from damage under ischemia/reperfusion [60,61].

Supplemental ginger oil not only promoted the phagocytic activity of heterophils per se [62] but also increased the percentage of circulatory heterophils in broilers, suggesting its enhanced innate immunity against pathogens [50]. Numerous reports suggest that ginger may act as a natural immunity booster. For instance, a study demonstrated that red ginger powder strengthened the immune system of broilers by upregulating intestinal IgA and splenic IgG levels and decreasing spleen, cecum, and ileum damage under *Salmonella* enteritidis [63]. In other studies, aqueous ginger extracts were shown to enhance humoral immunity in humans, as demonstrated by an increased serum IgM level in non-smoker males, which may lead to a greater antibody response against infections [64]. In broilers, supplemental ginger extracts were shown to raise antibody titers against sheep red blood cells (SRBC) [65], infectious bronchitis (IB), and Newcastle disease (ND), as well as avian influenza (AI, H5N8) [66]. These results undeniably indicate that ginger and its extracts act as potential immunostimulatory agents to enhance humoral immunity during vaccination or infection.

### 2.2. Turmeric and Its Active Compounds

The turmeric (*Curcuma longa*) plant is a member of the ginger family, Zingiberaceae, and its rhizome has long been used in traditional Chinese medicine to treat chronic illnesses such as metabolic derangements and cardiovascular diseases [67]. Curcumin, a yellow polyphenolic pigment and bioactive constituent derived from the rhizome of turmeric [68], has drawn significant attention due to its potential therapeutic effects. Studies have documented the potent anti-inflammatory, antioxidant, anti-cancer, and anti-diabetic effects of curcumin, making it a promising compound for the prevention and treatment of various diseases (Table 2) [68,69,70,71].

Curcumin also possesses anti-microbial properties, including against bacteria, protozoa, viruses, and fungi, and it even has immunomodulatory functions [69]. Its anti-bacterial functions have been shown to be effective against various Gram-negative and -positive bacteria, including *A. baumannii*, *E. faecalis*, *K. pneumoniae*, *Bacillus subtilis* (*B. subtilis*), *Staphylococcus epidermidis*, *Bacillus cereus* (*B. cereus*), *Listeria innocua*, *Helicobacter pylori* (*H. pylori*), *Salmonella enterica* serotype *Typhimurium*, and *Streptococcus mutans*, *Streptococcus pyogenes*, *S. aureus*, *Enterococcus faecalis*, and *Pseudomonas aeruginosa* [70]. The methanol extracted from rhizomes also have potent effects against *Escherichia coli*, *Staphylococcus aureus*, *Salmonella typhi*, and *Candida albicans* [71]. Curcumin has been shown to alleviate some autoimmune diseases by regulating inflammatory cytokines and associated JAK, AP-1 (activator protein 1), and NF-κB signaling in immune cells [72].

#### 2.2.1. Effects of Turmeric and Its Active Compounds on the Growth Performance of Broiler Chickens

Several studies showed that supplemental turmeric improved body weight gain [73], feed intake, and/or FCR in broilers [73,74]. A study even reported that cooked turmeric rhizome meal can enhance nutritional value and improve liveweight and carcass quality in broilers [75]. Moreover, a dietary supplementation of turmeric was shown to relieve the impact of aflatoxin in broilers and even improved body weight gain and FCR [76]. In addition, supplemental turmeric powder potentially affected the behaviors of broiler chickens under stress, leading to improved growth performance overall [77]. These affected behaviors in a high stocking density environment included drinking, feeding, crouching, feather-dressing, standing, and walking. Similar effects were also observed in ducks with supplemental curcumin in their diets [78]. Moreover, curcumin enhances intestinal morphology in broiler chickens, as observed by increased villus heights and villus height-to-crypt-depth ratios in all the segments of the intestines [79,80].

#### 2.2.2. The Anti-Inflammatory and Immunostimulatory Properties of Turmeric and Its Active Compounds

Despite few studies on the topic, there have been reports on the promising effects of turmeric and its active compounds as an anti-inflammatory and immunostimulatory agent in broilers with CP infections. Birds treated with turmeric could withstand the adverse effects of CP, as evidenced by improved body weight [81], FCR [82], and decreased intestinal lesions [81]. These alleviating effects were attributed to decreased serum levels of α-toxins and Net B-toxins produced by CP and the antibodies elicited against the toxins, as well as alleviated gene expressions, which encoded pro-inflammatory cytokines and chemokines in the intestine and spleen—presumptive due to lower CP colonization in the intestine and/or faster degradation of Net B-toxins in the circulation [81]. Diminished serum α-toxin and Net B-toxin levels were thought to be the key virulence factor in NE [21]. As discussed above, coccidia infections work synergistically with CP, causing clinical NE occurrence in chickens [16]. Supplemental turmeric or curcumin reduces the impacts of *Eimeria* infection on growth efficiency and intestinal health in broilers [83,84] through anti-inflammatory and immunostimulatory regulation. A study with micronized curcumin inclusion in a diet reported dramatically alleviated inflammation in an *Eimeria* infection, as shown by decreased intestinal TNF-α and IL-10 levels [84]; subsequently, the occurrence of clinical NE was notably prevented. However, the effect of curcumin is IL-10-dependent; it works synergistically with IL-10 to inhibit NF-κB signals in order to protect intestinal epithelial cells from inflammation [85]. These results undeniably posit turmeric and its active compounds as effective agents to combat NE in broilers through anti-inflammation and immunomodulation, in order to sustain their growth performance.

Several studies have found curcumin to have substantial anti-inflammatory and antioxidant effects by inhibiting MAPK [86,87], leading to the downregulation of pro-inflammatory IL-1β [88], TNF-α, IL-6 [77,89], and IL-2 [77]. Anti-inflammatory effects of curcumin were also reported in laying chickens under adverse impacts by heat stress, in which curcumin downregulated TLR and NF-κB signaling and inflammation in the liver, leading to a decrease of pro-inflammatory cytokine levels in the circulation [90].

Curcumin promotes intestinal barrier permeability by protecting tight junction proteins such claudins and occludins [91]. The reduction in TNF-α, IL-6, and IL-β by curcumin leads to the upregulation of intestinal tight junction proteins such as ZO-1, occludins, and claudin-1 [84], thereby leading to a faster recovery of intestinal mucosal layers [92]. In the case of a *C. jejuni* infection, curcumin treatment alleviated the inflammatory response and upregulated intestinal tight junction protein expressions [93].

Turmeric and curcumin also exert immuno-boosting effects on the humoral immunity of broilers. Adding turmeric in the diet of broilers increased serum IgA, IgM, and IgG levels [94], as well as specific antibody titers against SRBC [95] and Newcastle Disease (ND) [94]. Dietary curcumin treatment improved serum IgG, IgM, and IgA levels [96,97], as well as antibody titers against infectious bursal disease (IBD) [98]. These results support turmeric and its active compounds as potential natural immunostimulatory agents for vaccination in broiler production.

### 2.3. Alpinia spp. Extracts and Their Active Compounds

*Alpinia* spp. belongs to the ginger family, Zingiberaceae, and its extracts have been found to contain active compounds for therapeutic properties [99], including antioxidant, anti-inflammatory, and anti-microbial activities (Table 3) [100,101,102,103,104,105,106,107,108,109,110,111,112,113].

5,6-dehydrokawain (DK) and dihydro-5,6-dehydrokawain (DDK), found in the rhizomes and leaves of *Alpinia zerumbet*, exhibit antioxidant [101] characteristics and profoundly inhibit collagenase, elastase, hyaluronidase, and tyrosinase activity [100]. Chloroform and methanolic extracts of the flowers of *Alpinia zerumbet* hold promising effects as a natural remedy for combating tumors and oxidative stress-related diseases [102]. These strong antioxidant properties reside in phenolic compounds from the rhizomes and leaves of *A. zerumbet* and *A. conchigera*, including curcumin, 6-gingerol, eugenol, and vitamin C [103]. 1,8-cineole, α-farnesene, α-cadinene, -terpineol, α-bergamotene, and globulol are among the essential oils found in the rhizomes of *Alpinia* spp., with potent anti-bacterial effects against Gram-positive and -negative bacteria, in addition to possessing anti-fungal activities [104,105]. Cineole also has an antioxidant effect [114]. *Staphylococcus aureus*, *E. coli*, and *Shigella fleneri* are sensitive to bioactive compounds such as 4-terpineol, 1,8-cineole, γ-terpinolene, sabinene, and monoterpenes extracted from the leaves of *Alpinia* spp. [106,108]. Additionally, the flowers of *Alpinia* species, which contain high concentrations of triterpenoids, flavanoids, and alkaloids, have been shown to be effective against bacteria that cause sinusitis, such as *Porphyromonas gingivalis*, *Fusobacterium nucleatum*, *Fusobacterium necrophorum*, and *Prevotella intermedia* [107]. The ethanolic extracts from the dried seeds of *A. katsumadai* have anti-adhesive effects against *Campylobacter jejuni* [109], while methanolic extracts from flowers and rhizomes are effective against *Micrococcus luteus* and have been used to treat intestinal infections and other diseases [110]. The palmitic acid present in the leaf extracts of *Alpinia zerumbet* and *Alpinia pupurpurata* also have anti-fungal properties against *Cryptococcus neoformans*, *Fonsecaea pedrosoi*, *Trichophytoon rubrum*, *Microsporium canis*, and *M. gypseum* [111]. *Alpinia zerumbet* leaves and rhizomes can be hydro-distilled to produce zerumbone [112], which has been proven to possess anti-inflammatory and protective effects against dietary contaminants that cause hepatotoxicity [113].

#### 2.3.1. Effects of *Alpinia* spp. on the Growth Performance of Broiler Chickens

*Alpinia* spp.—specifically, its active components— has been proven to improve the growth performance of broilers when used as a feed supplement. The active ingredient 1-8-cineole in *Alpinia* spp. increased average daily gain (ADG) and improved FCR in broilers [115,116] even under stress conditions [116]. The intestinal morphology of broilers was differentially improved by 1,8-cineole, including increased villus heights and the ratio of villus height to crypt depth, thereby encouraging absorption and leading to better growth performance [115]. The curcumin contained in *Alpinia* spp. was shown to stimulate digestion, increase feed intake, and boost growth performances [117]. However, one study showed no beneficial effects from 1,8-cineole on production efficiency in laying chickens, and the laying chickens even had poor feed consumption and FCR, which in turn impaired egg production and other qualities including egg weight, shell strength, and eggshell thickness [118]. Furthermore, the addition of *Alpinia* spp. extracts to drinking water had no significant influences on growth performance [119,120].

#### 2.3.2. The Anti-Inflammatory and Immunostimulatory Properties of *Alpinia* spp. and Its Active Compounds

Very few studies have been performed to examine the anti-inflammatory effects of *Alpinia* spp. on broiler chickens with CP infections. However, some related studies showed that *Alpinia* spp. and its active compounds possess potential anti-inflammatory activities by targeting the inflammatory response (Figure 2). 1,8-cineole in *Alpinia* spp. was shown to possess anti-inflammatory and immunomodulatory functions by downregulating TNF-α, IL-1β, NF-κB, and the activation of ERK (extracellular signal-regulated kinases) in an animal model of acute pulmonary inflammation [104,121]. This likely occurred because 1,8-cineol suppressed cytokine production, including TNF-α, IL-1β, IL-6, and IL-8, in cultured human lymphocytes and monocytes [122]. Additionally, the γ-terpinene of *Alpinia* spp. with anti-inflammatory functions has been used as a therapeutic agent to suppress IL-1β and TNF-α production and reduce leukocyte migration under inflammatory conditions, such as peritonitis acute lung injury [123]. The zerumbone essential oil derived from *Alpinia* spp. also has anti-inflammatory effects that can suppress pro-inflammatory cytokine production [124] by promoting IκBα activation and inhibiting the IKK/NF-κβ pathway [125,126,127]. These modulatory effects provide potential therapeutic benefits under inflammatory conditions.

Similar to other ginger species, the active ingredient of *A. zerumbet*, 1-8 cineole, promotes humoral immunity in broilers, as shown by increased serum IgA, IgG, and IgM levels, which in turn benefits intestinal defense and provides resistance to pathogen infection [115].

### 2.4. Origanum Vulgare and Its Active Compounds

*Origanum vulgare*, widely found in temperate Asian regions, has been traditionally used in medicine for its therapeutic properties [128]. Several studies have demonstrated the antioxidant, antibacterial, and anti-fungal functions of *Origanum vulgare* and its bioactive compounds (Table 4) [129,130,131,132,133,134,135,136,137].

A recent study found that *Origanum vulgare* contains abundant rosmarinic acid, which has a high antioxidant activity [129] and is also found in other members of the *Lamiaceae* family, including rosemary and peppermint [138]. Dried aerial parts also contain thymol, γ-terpinene, carvacrol, p-cymene, and elemol, which have been proven to have a strong antioxidant effect [130]. The flowers of *Origanum vulgare* are also enriched with thymol and carvacrol [131] and sesquiterpene hydrocarbon-germacrene and €-caryophyllene monoterpene hydrocarbon-sabinene and oxygen-containing monoterpenes-terpinen-4-ol [132]. These active compounds thus account for why a dietary supplement of *Origanum vulgare* was found to possess greater antioxidant activity than Vitamin E in diets for broilers [130,139,140,141] and pigs [142].

Rosmarinic acid, an active compound present in the areal part of *Origanum vulgare*, also possesses anti-microbial functions against *Salmonella* enteritidis and *Aspergillus niger* [133]. Carvacrol, thymol, and y-terpinene from dried leaves and aerial parts possess antibacterial activities [134] against Gram-positive and -negative bacteria such as *Brochothrix thermosphacta*, *E. coli.*, *Listeria innocua*, *Listeria monocytogenes*, *Pseudomonas putida*, *Salmonella typhimurium*, and *Shewanella putrefaciens* [135]. Additionally, carvacrol is a clear yellowish liquid with an antibacterial activity against *Listeria monocytogenes*, *Staphylococcus*, *Bacillus subtilis*, *Bacillus cereus*, *E. coli*, *Salmonella entirica*, and *Klesialla* [136,143]. Carvacrol and thymol were found to be effective against *C. perfringens* spore germination and outgrowth in turkey meat during chilling and storage [144]. Interestingly, the aqueous extract from *Origanum vulgare* was found to possess antibacterial effects that benefitted chicken health [145]. Carvacrol and thymol, as the main compounds derived from *Origanum vulgare* leaves, possess anti-fungal activity against *C. albicans* and *C. glabrata* [137].

#### 2.4.1. Effects of *Origanum vulgare* on the Growth Performance of Broiler Chickens

Several studies have demonstrated the effect of supplemental *Origanum vulgare* in various forms. Supplemental ground oregano leaves increased the overall growth performance parameters of broilers [140,146,147]. In other instances, the effect of the supplemental aqueous extract of *Origanum vulgare* was limited, merely increasing body weight gain but not FCR [139,148], whereas feed intake, body weight gain, and FCR were improved by dietary inclusion of an essential oil derived from *Origanum vulgare* [149,150,151]. Some of the studies concluded that oregano essential oil was a potential replacement for AGPs, since the growth performance of broilers with supplemental oregano essential oil was even better than those with AGPs [140,149].

*Origanum vulgare* supplements, regardless of their form, are effective as growth promoters in broilers and layers [152,153,154,155]. Dietary rosmarinic acid supplementation was found to be effective in improving body weight gain and FCR in broiler chickens [141]. Thymol and carvacrol, the major essential oils derived from *Origanum vulgare*, were also effective in promoting the growth performance of broilers [156,157,158,159]. Compounding a mixture of phytogenics, such as carvacrol and thymol, with cinnamaldehyde and capsicum [160,161] was shown to improve intestinal morphology, thus resulting in a better absorption process and promoting production performances in both laying hens [162] and broilers [159]. Another study further demonstrated that compounding the thymol and carvacrol derived from *Origanum vulgare* as a dietary supplement had a protective effect on broilers against pathogenic *C. perfringens* [163], as they relieved adverse effects and improved growth performance [164].

#### 2.4.2. The Anti-Inflammatory and Immunostimulatory Properties of *Origanum vulgare* and Its Active Compounds

Several studies demonstrated that the combination of thymol and carvacrol as a dietary supplement can lower *C. perfringens’* virulence factors and ultimately result in relieved gut lesions and NE severity in broilers [157,163,164]. Supplemental oregano essential oil even effectively ameliorates the negative effect of coccidia infection, the major predisposing factors of NE. A dietary supplementation of oregano essential oil increased the expression of zonula occludens-1 (ZO-1) in broilers with coccidiosis [165]. Moreover, when challenged with LPS, carvacrol essential oil, via oral administration, also profoundly upregulated the intestinal expression of tight junction protein occludins, claudin-1, claudin-5, ZO-1, and ZO-2 in broilers [166]. The dietary supplementation of blended oregano essential oil, mainly containing carvacrol and thymol, was shown to benefit intestinal integrity by upregulating tight junction protein expressions in all segments of the intestine in pigs [167].

The inflammation-protective functions of the essential oil from *Origanum vulgare* were confirmed in some studies. The anti-inflammatory effects of carvacrol were demonstrated via inhibition of MAPK and NF-κB pathways and the downstream of inflammatory cytokines in Wistar rats with renal injuries [168,169]. An oral administration of carvacrol essential oil suppressed NF-κB expression, leading to the downregulation of intestinal TNF-α, IL-1β, IL-6, and IL-8 in broilers challenged with LPS [170]. The suppression of NF-κB expression and downstream pro-inflammatory cytokine production by thymol and carvacrol operated in a synergistic manner [159]. The anti-inflammatory effect of oregano essential oil was also observed in broilers infected with *C. perfringens* [157,164].

Oregano essential oils also have immunostimulatory effects that increase antibody titers in broilers. The supplementation of oregano essential oil increased the level of intestinal sIgA [148,149] and serum IgE and IgG [158]. The dietary supplementation of a thymol and carvacrol mixture even elevated serum IgA levels in broilers with CP infections and promoted antibody titers against SRBC [156] and ND after vaccination [161].

### 2.5. Clove Extracts and Its Active Compounds

Cloves are aromatic flowers from *Syzygium aromaticum* (L.) Merr. & L.M. Perry, a tree native to Indonesia. They have been recognized for their therapeutic effects against various diseases in China and in Western countries [171]. The therapeutic properties of cloves can be attributed to the richest bioactive compound, eugenol, which possesses anti-inflammatory, antioxidant, and antibacterial functions (Table 5) [46,172,173,174].

Clove extracts were discovered to contain potential phytogenics with anti-inflammatory [172], anti-microbial [46,173], and antioxidant effects [174]. Eugenol is the most important component of cloves. It modulates inflammatory response via inhibition of the NF-κB pathway [175]. The antioxidant property of cloves is associated with high phenol and flavonoid content, which results in cloves possessing a high proportion of antioxidant components compared to other spices [176]. Other studies have also demonstrated that cloves can suppress bacterial proliferation, such as in the cases of *Escherichia coli*, *Staphylococcus aureus*, and *Pseudomonas aeruginosa* [46,173]. In addition, cloves exert virulence-modulating effects by perturbing the expression of virulence-associated genes for synthesis, such as the flagellin genes in *Campylobacter jejuni*, a disease-causing gastro-intestinal infection [173]. The antibacterial effects of clove extracts against CP were also reported in broilers [46,177].

#### 2.5.1. Effects of Cloves Extracts on the Growth Performance of Broilers

Dietary clove supplementation has been shown to improve growth performances in broilers [178]. The broilers with CP infections that received a diet containing cloves showed relieved intestinal damage in addition to improved body weight gain [177] and FCR [179]. Parts of the improved performance were attributed to intestinal morphology and microbiota, mainly by the proliferation of *Lactobacillus* spp., which improves the villus length of the small intestine, thereby favoring better absorption [179,180].

The beneficial effect of supplemental cloves on growth performance depends on the inclusion rate. Increasing the amount of clove powder in the diet from 30 to 60 g/kg decelerated the growth performance in broilers [181]. The inclusion of cloves at a 1% level increased feed intake, average weight gain, FCR, and carcass weight, whereas a 1.5% inclusion rate had no such effects on growth parameters [180]. However, the body weight gain of broilers decreased with increasing levels of clove seed inclusion in the diet [182]. Some reports also showed no significant effects on average body weight gain if supplied at higher clove concentrations [183]. The reduction in growth parameters caused by higher doses of cloves was attributed to their lower palatability due to a high amount of eugenol [184]. Furthermore, higher levels of supplemental cloves may increase the activity of aspartate aminotransferase (AST) [185], which is associated with liver damage [186]. Higher AST levels may lead to indistinguishable changes in the body weight gain of individual broilers [187]. Different results in quails showed that increasing the amount of clove oil in the diet improved body weight gain, feed intake, and FCR from 1 to 6 weeks of age [188]. Despite the discrepant results, the improved growth parameters in healthy or CP-infected broilers were attributed to their anti-microbial activity, increased digestive enzyme secretion, and improved intestinal morphology, thus enhancing intestinal digestion and absorption [177,179,189,190].

#### 2.5.2. The Anti-Inflammatory Effects of Clove and Its Active Compounds

Eugenol, the main bioactive compound in clove oil, suppressed intestinal inflammation and serum inflammatory cytokine levels by inhibiting NF-κB signaling through NF-κB phosphorylation, leading to the upregulation of tight junction proteins such as ZO-1, Occludin, and Claudin-1 in the jejunum of piglets infected with a transmissible gastro-intestinal enteritis virus [191], which in turn fortified the interaction among intestinal epithelial cells and stabilized their integrity, resulting in the formation of a solid physical barrier during infection [192]. The anti-inflammatory effects of clove extracts were also observed in broilers, even in those with a CP [157] or *Salmonella* infection [193], in which TNF-α expression [157] and NF-κB signaling were suppressed, and the expression of intestinal tight junction proteins, ZO-1, claudin, and occludin, as well as junction adhesion molecule JAM-2, were sustained [193,194]. The inhibition of NF-κB pathways by clove extracts not only downregulates intestinal IL-1β, TNF-α, IL-6, IL-8, and IL-10 during infection [193,195,196] but also hampers peripheral macrophage functions, leading to the suppressed production of proinflammatory cytokines [197].

Eugenol has been demonstrated as being a potent compound that stimulates humoral immunity, even under stress circumstances. In the case of pigs infected with a transmissible gastroenteritis virus, eugenol elevated the levels of serum IgG [191].

## 3. Proposed Mechanisms for the Phytogenics of Ginger, *Origanum vulgare*, and Cloves against Inflammation to Improve Intestinal Integrity

Phytogenics and their active compounds primarily operate by inhibiting NF-κκB and MAPK pathways to downregulate pro-inflammatory cytokines and increase tight junction proteins and intestinal integrity (Figure 2). Moreover, phytogenics can act as immunomodulators to regulate innate and humoral immunity, leading to beneficial effects for growth performance. These mechanisms suggest that phytogenics can alleviate systemic and intestinal inflammation, modulate innate and humoral immunity, and improve gut barrier function to benefit broiler health and growth performance.

## 4. The Toxicity and Safety of Ginger, *Origanum vulgare*, and *Syzygium aromaticum* as Potential Feed Supplements

Despite the many advantageous effects of phytogenics as feed supplements, there are still some concerns about their safety, side effects, and unfavorable odor and flavors, not only for animal health but also for product quality [198,199]. Consequently, evaluations regarding levels of supplemental phytogenics, in order to avoid possible toxicity and undesirable meat quality, are required.

Incorporating ginger extracts mainly containing 6-gingerol, 8-gingerol, 10-gingerol, and 6-shogaol into a diet at the maximal dosage of 2000 mg/kg body weight in rats showed no mortality and clinical signs of toxicity [200]. Therefore, the LD50 (Lethal Dosage 50%) is regarded as >2000 mg/kg in rats. According to the OSHA’s HCS (Occupational Safety and Health Act, Hazard Communication Standard) in the U.S., the LD50 of an oral administration of 6-gingerol, the major active compound in ginger, is 250 mg/kg in mice. Comparable results in broilers showed that the dietary inclusion of dried ground red ginger at 1.5% improved growth performance, despite noticeable morphological changes in the bird’s internal organs—such as edema, necrosis, and inflammation in the muscle, liver, and kidneys—while up to 2.0% had no positive effects on growth performance, rather presenting an exacerbated inflammatory status and necrosis [201]. Ginger may contain abundant sesquiterpenes, which are thought to exert toxic effects on animals [202]. However, sesquiterpenes could be beneficial, as they possess protective, antioxidant, and anti-inflammatory effects against ethanol-induced gastric mucosa damage [203].

In a one-dose treatment, followed by a 14-day observation for acute toxicity, both rats and mice receiving oral administrations of 5000 mg/kg of a curcuminoid–essential oil complex (CEC) from turmeric showed no mortality and no abnormalities of pathological parameters [204]. In a repeated oral administration of CEC for 90 days, an amount of up to 1000 mg/kg body weight did not induce any noticeable toxic effects in rats. The OSHA’s HCS defined the LD50 of an oral dosing of curcumin as being >2000 mg/kg in rats.

A 90-day oral toxicity study of oregano essential oil in rats revealed that up to 200 mg/kg had no adverse effects on food/water consumption, body weight, hematology, biochemistry, organ weight, and histopathology [205]. A higher dose of *Origanum vulgare* extracts at 400 mg/kg, however, caused hepatotoxicity, including cellular degeneration and distortion, inflammatory infiltration, cytoplasmic vacuolization, sinusoidal congestion, and apoptosis in a 7-day study in mice [206].

Oral administrations of up to 60 mg/kg with hexane extracts from the flower buds of *Syzygium aromaticum* had no systemic toxicity in a 35-day study in mice [207]. However, a 60 mg/kg dose exerted adverse effects on the sperm quality of male mice, as shown by degenerative changes in the seminiferous tubules in association with decreased spermiogenesis [207]. In domestic animal cases, a dietary supplementation of eugenol from cloves at 0.5% and 1.0% levels had no adverse effects on the growth performance and immune organs in male layer chickens, while 1.5% of eugenol significantly decreased thymus weight—but not in the bursa of Fabricius, spleen, and liver [208]. The OSHA’s HCS defined the LD50 of an oral administration of eugenol as 2130 mg/kg in guinea pigs, 1930 mg/kg in rats, and 3000 mg/kg in mice.

## 5. Conclusions

These consolidated findings suggest that phytogenics are a practical and effective option for addressing the problem of AMR, and they present an alternative to AGPs in the prevention of infectious diseases in broilers, particularly NE. It has been discovered that phytogenics boost absorption and digestion by improving intestinal morphology. In addition, the antibacterial property of phytogenics can restrain pathogenic bacteria in the gut, lowering the risk of infection and enhancing the balance of gut microbiota overall. Past studies with phytogenics in broilers demonstrated encouraging results in the improvement of gut health and enhancement of the immune system under normal conditions, which were eventually effective in broilers with CP infections as well. These active substances, such as 6-shagol, gingerol, curcumin, 1-8-cineole, zerumbone, rosmarinic acid, thymol, carvacrol, and eugenol, offer a mechanism for squelching pathogen infection by targeting NF-κB and MAPK pathways. These pathways modulate immune response and inflammation in order to alleviate the symptoms of NE and thus enhance overall gut health in broilers. Overall, incorporating phytogenics into poultry diets is promising for approaches aiming to promote gut health, optimize immune function, and improve growth performance in broilers while reducing the use of antibiotics.

## Figures and Tables

**Table 2:** Turmeric extract plant parts, active compounds, and biological properties.

Bioactive Components or Extracts	Parts of the Plant for Extraction	Functional Property	References
Curcumin	Rhizomes	Effective for cardiovascular diseases, diabetes, and cancers, with neuroprotection, anti-inflammatory, and antioxidant functions	[68,69]
		Anti-bacterial, anti-protozoal, antiviral, immunomodulatory, and anti-fungal effects	[69]
		Antibacterial effects against various Gram-negative and -positive bacteria, including *A. baumannii*, *E. faecalis*, *K. pneumoniae*, *P. aeruginosa*, *Bacillus subtilis* (*B. subtilis*), *Staphylococcus epidermidis*, *Bacillus cereus* (*B. cereus*), *Listeria innocua*, *Streptococcus pyogenes*, *S. aureus*, *Helicobacter pylori* (*H. pylori*), *Escherichia coli* (*E. coli*), *Salmonella enterica* serotype *Typhimurium*, and *Streptococcus mutants*	[70]
		Effective against *Streptococcus pyogenes*, *S. aureus*, *Enterococcus faecalis*, and *Pseudomonas aeruginosa*	[71]

**Table 3:** Plant parts, active compounds, and biological properties of *Alpinia* spp. extracts.

Bioactive Components or Extracts	Parts of Plants for Extraction	Functional Property	References
5,6-dehydrokawain (DK), dihydro-5,6-dehydrokawain (DDK)	Rhizomes	Antioxidant properties and is an effective inhibitor of collagenase, elastase, hyaluronidase, and tyrosinase	[100]
	Leaves	Possesses the highest antioxidant properties and anti-aging effects	[101]
CH_2_Cl_2_ and MeOH extracts	Flowers	Possesses a higher potentiality of anti-tumor effects and antioxidant properties by upregulating superoxide dismutase (SOD) and catalase (CAT) in the liver	[102]
Phenolic compounds such as curcumin, 6-gingerol, eugenol, and vitamin C	Rhizomes	Antioxidant properties	[103]
Essential oils such as 1,8-cineole, α-farnesene, γ-cadinene, α-terpineol, α-bergamotene, and globulol	Rhizomes	Anti-microbial properties against Gram-positive bacteria in addition to anti-fungal activities.	[104,105]
4-terpineol, 1,8-cineole, γ-terpinolene, sabinene, and monoterpenes	Leaves	Effective against *Staphylococcus aureus* and *E. coli*	[106]
Triterpenoids, flavonoids, alkaloids	Flowers	Effective against bacterial pathogens causing sinusitis, including *Porphyromonas gingivalis*, *Fusobacterium nucleatum*, *Fusobacterium necrophorum*, *Streptococcus pneumoniae*, and *Prevotella intermedia.*	[107]
Hydroxybenzoic acids, hydroxycinnamic acids, and flavonoid extracts	Fresh rhizomes	Effective against *Eschiricia coli*, *Staphylococcus aureus*, and *Shigella fleneri.*	[108]
*Ethanol* extracts	Dried seeds of *A. katsumadai*	Anti-adhesive effects against *Campylobacter jejuni*	[109]
*Methanol extracts*	Flowers and rhizomes	Effective against *Micrococcus luteus*, and treatment for intestinal infections and other diseases.	[110]
Palmitic acid (*n*-hexadecanoic acid)	Leaves from adult plants	Anti-fungal activities against *Cryptococcus neoformans*, *Fonsecaea pedrosoi*, *Trichophytoon rubrum*, *Microsporium canis* and *M. gypseum*	[111]
Zerumbone, a-caryophyllene, and camphene	Hydrodistillation of *Alpinia zerumbet* leaves and rhizomes.	Effective against food contaminants that cause hepatotoxicity; possesses anti-inflammatory properties	[112,113]

**Table 4:** Plant parts, active compounds, and biological properties of extracts of *Origanum vulgare*.

Bioactive Components or Extracts	Parts of the Plant for Extraction	Functional Property	References
Rosmarinic acid	Dried leaves	Antioxidant activities	[129,130]
Thymol, γ-terpinene, carvacrol, p-cymene, and elemol	Dried aerial parts	A stronger antioxidant activity than Vitamin E	[130]
Thymol and carvacrol	Flowers	An antioxidant function	[131]
Sesquiterpene hydrocarbon-germacrene, (e)-caryophyllene monoterpene hydrocarbon-sabinene, and oxygen-containing monoterpenes-terpinen-4-ol	Flowers	An antioxidant function	[132]
Rosmarinic and chlorogenic acids	Aerial part	Anti-microbial properties against *Salmonella enteritidis* and *Aspergillus niger* with hepatoprotective effects	[133]
Thymol and carvacrol	Flowers	Effective against Gram-negative bacteria, with anti-fungal properties	[131]
Carvacrol, β-fenchyl alcohol, thymol, and γ-terpinene	Dried aerial part	Antibacterial properties against Gram-positive and -negative strain bacteria, *Brochothrix thermosphacta*, *E. coli*, *Listeria innocua Listeria monocytogenes*, *Pseudomonas putida*, *Salmonella typhimurium*, and *Shewanella putrefaciens*	[134,135]
Carvacrol	Aerial parts	Antibacterial effects against *Staphyloccus aureus*	[136]
Carvacrol (the main compound) and thymol	Leaves	Fungicidal activities against *C. albicans* and *C. glabrata*	[137]

**Table 5:** Plant parts (*Syzygium aromaticum*), active compounds, and biological properties of the extracts of Cloves.

Bioactive Components of Cloves	Biological Property	References
Eugenol	Anti-inflammatory effects	[172]
	Antibacterial properties against *E. coli*, *Staphylococcus aureus*, and *Pseudomonas aeruginosa*, *Clostridium perfringens*, and *Campylobacter jejuni*	[46,173]
	Antioxidant properties	[174]

## Data Availability

Not applicable.
